# Determination of the molecular mechanism by which macrophages and γδ-T cells contribute to ZOL-induced ONJ

**DOI:** 10.18632/aging.104006

**Published:** 2020-10-25

**Authors:** Xingzhou Qu, Zhen Wang, Tian Zhou, Liancheng Shan

**Affiliations:** 1 Department of Oral and Maxillofacial-Head Neck Oncology, Ninth People’s Hospital, College of Stomatology, Shanghai Jiaotong University, School of Medicine, Shanghai, 200011, China; 2 Department of Orthopedics, Tongren Hospital, Shanghai Jiaotong University School of Medicine, Shanghai, 200336, China

**Keywords:** γδ-T cells, macrophages, IFN-γ, osteoclasts, jaw necrosis

## Abstract

Objective: This study aims to explore the molecular mechanism of macrophages and γδ-T cells in the ZOL drug-induced osteonecrosis of jaws based on the IFN-γ involved osteoblast differentiation signaling pathway.

Results: The number and apoptotic rate of CD11b+Gr1hi cells and γδ-T cells in the ONJ group were significantly higher. The TNF-α, IL-1β, IFN-γ, CCL3, CCL4, IL-12 and IL-13 levels were significantly higher in the ONJ group. The expression of CTSK and FGFR3 was lower in the ONJ group, but was higher in the NF-κB and ERBB2IP group.

Conclusion: The proliferation of macrophages and γδ-T cells promote the inflammation in ZOL-induced jaw necrosis.

Methods: A total of 20 patients with osteonecrosis of the jaw from January 2016 to March 2018 were collected and assigned into the observation group, while 20 healthy subjects were assigned into the control group. Furthermore, 40 SD rats were selected and assigned into observation group, while 10 non-treatment SD rats were selected and assigned as controls. The distribution and proportion of CD11b+Gr1hi cells and γδ-T cells in the necrotic tissues of the jaw were analyzed. Then, the TNF-α, IL-1β, IFN-γ, CCL3, CCL4, IL-12 and IL-13 levels were measured. Afterwards, the expression of CTSK, FGFR3, NF-κB and ERBB2IP in the necrotic tissues of the jaw in the animal models were analyzed.

## INTRODUCTION

Medication-related osteonecrosis of the jaw (MRONJ) is a serious complication of jaw necrosis caused by the use of bisphosphonates (BPs) or other targeted drugs [1, 2]. Clinical studies have found that many drugs can cause jaw necrosis, and BP is the first drug found to cause MRONJ. Mounting reports have demonstrated that IFN- γ, macrophages and γδ-T cells have played potential roles in the pathophysiological process of MRONJ [3–5]. In general, ZOL can promote the TLR4-mediated M1 macrophage polarization in BP-induced jaw necrosis. In human patients with MRONJ lesions, the infiltrating γδ-T cells significantly increased, when compared to healthy subjects, indicating the critical role of γδ-T cells in the development of MRONJ. Furthermore, when these were pretreated with ZOL, the γδ-T cells co-cultured with the osteoclasts would secrete a large amount of IFN-γ due to the unknown mechanism. However, the exact mechanism of MRONJ still remains unclear.

Recent studies suggest that the pathogenesis of drug-induced jaw necrosis is correlated to osteoclast inhibition, microcirculation dysfunction, direct cytotoxicity, bacterial infection, and immune dysfunction [6]. Zoledronic acid (ZOL) is a potent bisphosphonate drug, which has been widely used in clinic. This can lead to bone resorption inhibition and angiogenesis inhibition, and subsequently lead to drug-induced jaw necrosis [7]. Cytokines, inflammatory chemokines and growth factors can not only regulate the function of immune cells, but also regulate the activity of osteoblasts and osteoclasts. Interferon-γ (IFN-γ) is an important immunoregulatory factor in the immune response. Studies have shown that IFN-γ participates in osteoblast differentiation, and can inhibit the formation of damaged cells *in vitro* [8]. Macrophages are important immune cells, and can secrete a variety of inflammatory factors, activate T cells, and stimulate specific immune effects [9, 10].

Furthermore, γδ-T cells are a kind of common immune barrier effector cells, which play an important role in immune activities. The present study aims to explore the molecular mechanism of macrophages and γδ-T cells that participating in ZOL drug-induced jaw necrosis through the IFN-γ-based signaling pathway involved in osteoblast differentiation.

## RESULTS

### Distribution of CD11b+Gr1hi cells and γδ-T cells in the MRONJ lesions of necrotic jaw tissues

**Figure 1:**
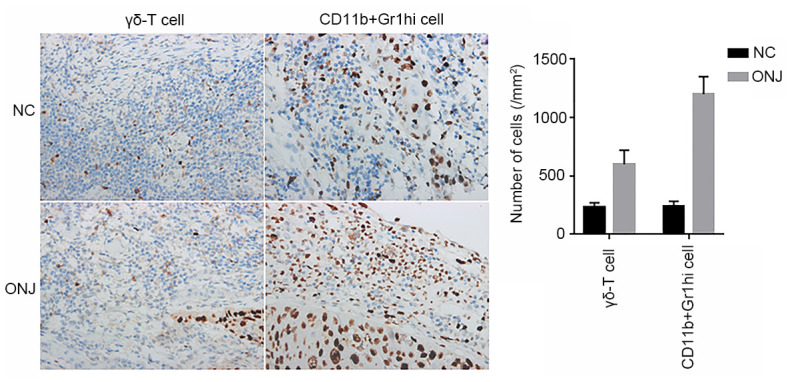
**The distribution of CD11b+Gr1hi cells and γδ-T cells in the necrotic tissue of the jaw.** ***P<0.001, compared with the NC group.

Compared with the number of CD11b+Gr1hi cells and γδ-T cells, the staining range was significantly larger in the ONJ group than in the NC group, and the number of cells was significantly higher in the ONJ group than in the NC group (*P*<0.05), indicating that CD11b+Gr1hi cells and γδ-T cells in necrotic tissues were at a large proliferation state, as shown in Figure 1.

### Proportional characteristics of CD11b+Gr1hi cells and γδ-T cells in necrotic jaw tissues of patients

**Figure 2:**
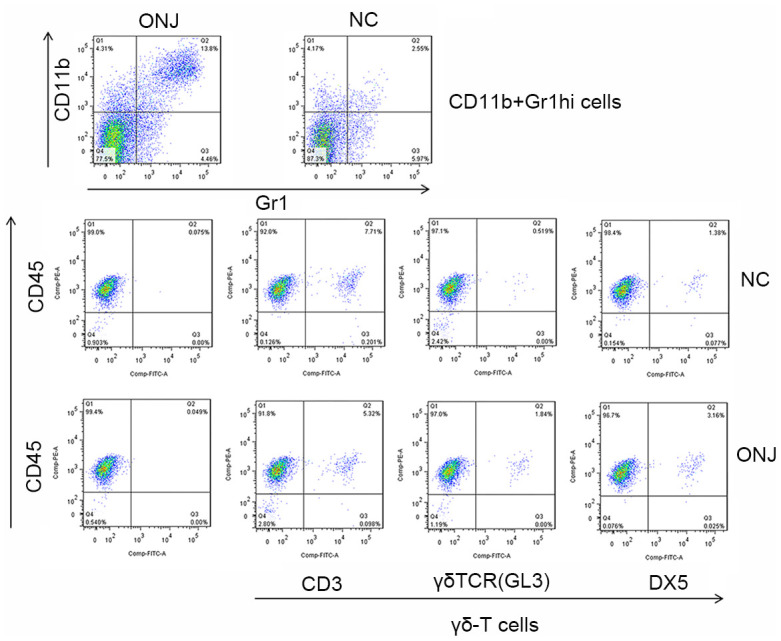
**The proportion of CD11b+Gr1hi cells and γδ-T cells in the necrotic jaw tissues of patients.**

The flow cytometry revealed that the apoptotic rate of CD11b+Gr1hi cells in the ONJ group was 13.8% higher than that in the NC group (2.55%) (*P*=0.004), and the apoptotic rate of γδ-T cells was 3.16% higher than that in the NC group (1.38%) (*P*=0.398). This shows that the apoptotic rate of CD11b+Gr1hi cells in necrotic tissues was higher, and the cell growth was abnormal, as shown in Figure 2.

### Characteristic of inflammatory factors TNF-α, IL-1β, CCL3, CCL4, IL-12, IL-13 and IFN-γ in the blood of patients

**Figure 3:**
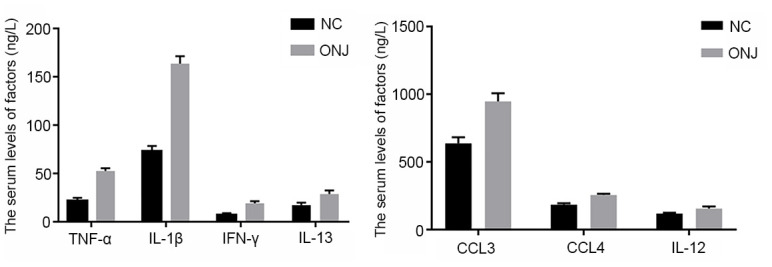
**The serum levels of inflammatory factors TNF-α, IL-1β, CCL3, CCL4, IL-12, IL-13 and IFN-γ.** ***P<0.001, compared with the NC group.

The serum levels of TNF-α, IL-1β, IFN-γ, CCL3, CCL4, IL-12 and IL-13 in the ONJ group were significantly higher than those in the NC group (*P*<0.05), indicating that the inflammatory reaction and immune response of patients were enhanced, as shown in Figure 3.

### Distribution of CD11b+Gr1hi cells and γδ-T cells in the necrotic tissues of rat jaw

**Figure 4:**
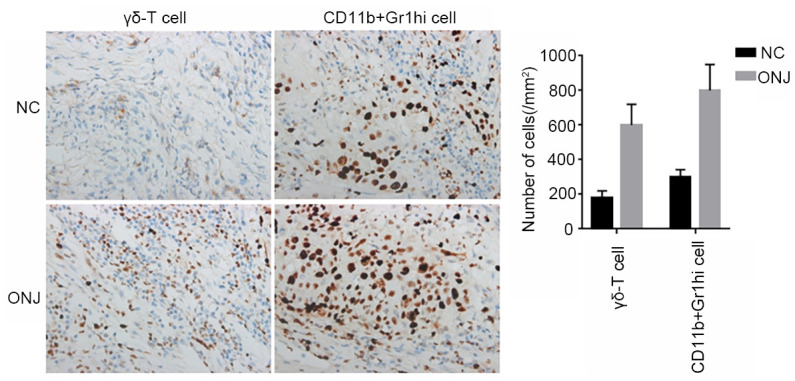
**The distribution of CD11b+Gr1hi cells and γδ-T cells in the necrotic tissues of rat jaws.**

Compared with the number of CD11b+Gr1hi cells and γδ-T cells, the number of cells in the ONJ group was significantly higher than that in the NC group (*P*<0.05), indicating that CD11b+Gr1hi cells and γδ-T cells in necrotic tissues were in a large proliferation state, as shown in Figure 4.

### Proportional characteristics of CD11b+Gr1hi cells and γδ-T cells in rat serum

**Figure 5:**
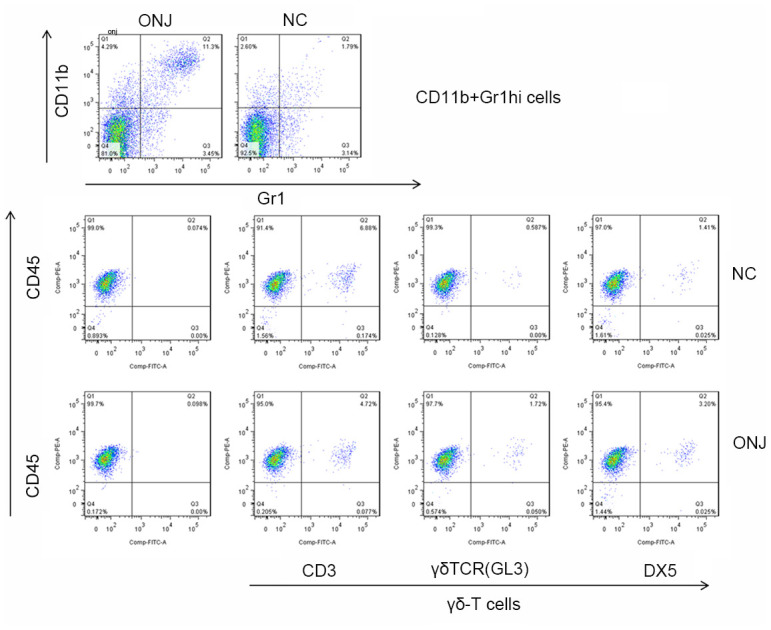
**The proportion of CD11b+Gr1hi cells and γδ-T cells in rat serum.**

The flow cytometry revealed that the apoptotic rate of CD11b+Gr1hi cells in the ONJ group was 11.3% higher than that in the NC group (1.79%) (*P*=0.007), and the apoptotic rate of γδ-T cells was 3.20% higher than that in the NC group (1.41%) (*P*=0.399). This shows that the apoptotic rate of CD11b+Gr1hi cells in necrotic tissues was higher, and the cell growth was abnormal, as shown in Figure 5.

### Characteristics of serum inflammatory factors TNF-α, IL-1β, CCL3, CCL4, IL-12, IL-13 and IFN-γ in rats

**Figure 6:**
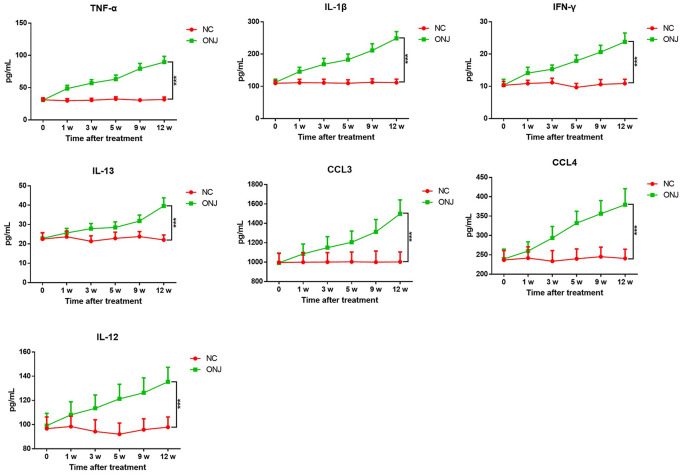
**The serum levels of inflammatory factors TNF-α, IL-1β, CCL3, CCL4, IL-12, IL-13 and IFN-γ in rats.**

The levels of TNF-α, IL-1β, IFN-γ, CCL3, CCL4, IL-12 and IL-13 in serum of rats in the ONJ group all gradually increased after treatment, and were significantly higher than those in the NC group (*P*<0.05), indicating that the inflammatory and immune responses of the model rats were enhanced, as shown in Figure 6.

### The expression of CTSK, FGFR3, NF-κB and ERBB2IP in jaw necrosis tissues

**Figure 7:**
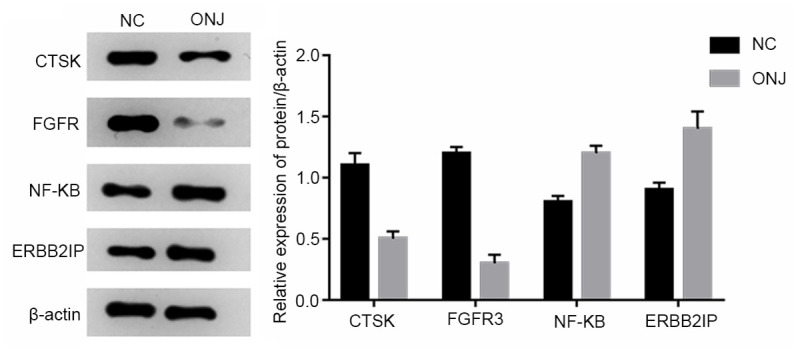
**The expression of CTSK, FGFR3, NF-κB and ERBB2IP in rat jaw necrosis tissues.**

The expression of CTSK and fibroblast growth factor R3 in the ONJ group was lower than that in the NC group, and the expression of NF-κB and ERBB2IP in the ONJ group was higher than that in the NC group. The difference was statistically significant (*P*<0.05). This indicated that osteoclast function was inhibited and bone metabolism was abnormal in the necrotic jaw of model rats, as shown in Figure 7.

## DISCUSSION

ZOL is a class of drugs that mainly act on osteoclasts, and inhibit bone resorption to maintain bone mineral density and strength. ZOL has been widely used to treat osteoporosis, Peggit's disease, multiple myeloma, breast cancer, lung cancer and other solid tumors with bone metastases. Under normal conditions, osteoblasts and osteoclasts can coordinate to induce the differentiation to maintain normal bone tissues. When ZOL inhibits the activity of osteoclasts, dead bone tissues cannot be removed in time, and excessively aggregates. Furthermore, the minor damage of aging bone tissues cannot be repaired, and the defense function against external trauma and infection invasion was reduced. This would induce the accumulation of inflammation damage, which is a vicious circle that leads to osteocyte necrosis, and the occurrence of jaw necrosis [11, 12].

Immune activity plays an important role in jaw necrosis. IFN-γ is mainly produced by Th cells, and plays a key role in promoting Thl polarization. The IFN-γ secreted by cells plays an important role in promoting Thl response, and inhibiting Th2 response [13]. IFN-γ has important clinical value in evaluating the function of the immune system and its therapeutic effect. Cellular immune response is an important way for the immune system to play an anti-tumor role. The polarization of Th cells to Thl or Th2 is regulated by a series of cytokines secreted by Th cells. Takayanagi reported an important antagonistic mechanism: activated T cells not only produce RANGKL-induced osteoclast formation and the differentiation under inflammatory conditions, but also expresses IFN-γ to inhibit RANKL [14]. In the present study, it was found that the number of γδ-T cells in the necrotic tissues of the jaw increased, with a large number of proliferations, suggesting that both play an important role in the necrosis of the jaw. The serum IFN-γ in the ONJ group had a high expression. Previous studies have shown that γδ-T cells play a key role in the early defensive immune response to pathogenic microorganism invasion, and express high levels of IFN-γ [15, 16]. Therefore, it was speculated that γδ-T cells may stimulate IFN-γ secretion by activating the TLR pathway, affecting the regulation of osteoclasts through the RANGKL signaling pathway, and ultimately inhibiting the function of osteoclasts and promoting jaw necrosis.

Macrophages can phagocytize and clean up foreign bodies and necrotic tissues, and release TNF-α, IL-1β and other cytokines to regulate tissue repair. M1 macrophages can be induced by IFN-γ to produce this [16, 17]. TNF-α is a cytokine with remarkable anti-tumor effects. In addition to its direct exertion killing effect on cancer cells, it can also effectively regulate the immune function of the body and enhance the clearance function of the immune system to malignant cells [18]. TNF-α concentration is low in normal conditions. A proper amount of TNF-α is necessary for the protection of the body, but excessive TNF-α can cause damage to the body. Inflammation is mediated by inflammatory factors. The typical inflammatory factors include IL-1β, IL-12 and IL-13. Cytokines can promote each other to cause an inflammatory cascade reaction, aggravate tissue damage, and participate in bone metabolism [19, 20]. Chemokines can regulate inflammation. CCL3 and CCL4 belong to the CC ligand family. In inflammation, macrophages can produce chemokines, which are highly expressed in infectious tissues. In the present study, the proliferation of macrophages in the ONJ group was fine, and the above-mentioned inflammatory-related factors had a high expression, suggesting that macrophages proliferated and secreted a large number of inflammatory factors, which promoted the local inflammation in patients with jaw necrosis, and aggravated tissue damage.

CTSK is a target protease for osteoporosis, which has attracted much attention in recent years. It was found that activated osteoclasts almost specifically express mature CTSK, accumulate in the lysosomal vesicles of osteoclasts, and are released into acidic microenvironment of extracellular absorption lacunae, especially degradable collagen type I [21, 22]. In the present study, the expression of CTSK in necrotic jaw tissues decreased, which resulted in the inhibition of osteoclasts, and affected the balance of bone metabolism. Fibroblast growth factor receptor 3 (FGFR3), which is a member of the Fibroblast Growth Factor (FGFRs) family, is a transmembrane receptor, which plays a negative regulatory role in cartilage development, and is closely correlated to the direct regulation of endochondral osteogenesis. The enhancement of the function of fibroblast growth factor R3 can inhibit the growth and apoptosis of chondrocytes. Fibroblast growth factor R3 participates in the maintenance of the stability of the adult temporomandibular articular cartilage. In the present study, the expression of fibroblast growth factor R3 in necrotic tissues of the jaw decreased, which may have some effect on necrosis of the jaw. NF-kB plays a role in the bone metabolism signaling pathway RANKL-RNAK-NF-kB, which is the central link of aseptic inflammation and osteoclast activation, and plays a positive regulatory role in regulating the number of osteoclasts [23]. In the present study, the expression of NF-kB protein in rat jaw necrosis tissues increased, suggesting that the positive regulation of osteoclasts was enhanced, and presumably, this was a compensatory response in the case of the massive osteonecrosis of jaw tissues, while the function of osteoclasts was still inhibited. Erbb2ip, which is also known as Erbin, was first discovered as an interacting protein of Erbb2 in 1997. This belongs to the LAP (leucine rich repeat and PDZ domain) protein family. Erbb2ip plays an important role in regulating cell proliferation and differentiation, cardiac development, myelin formation, and tumorigenesis and metastasis [24]. In the present study, the expression of ERbB2IP in necrotic jaw tissues increased, which was consistent with the proliferation of CD11b+Gr1hi cells and γδ-T cells.

In conclusion, the proliferation of macrophages and γδ-T cells in ZOL-induced jaw osteonecrosis promotes the expression of inflammatory factors, and IFN-γ, aggravates the inflammatory response and affects the differentiation pathway of osteoblasts involved by IFN-γ, thereby inhibiting the differentiation of osteoclasts. The present study also has some limitations. First, the investigators did not investigate the dynamic changes of CD11b+Gr1hi cells and γδ-T cells in patients, and the inflammatory factors. Second, the difference in CD11b+Gr1hi cells and γδ-T cells should be confirmed through more clinical samples. All these needs further researches to be confirmed.

## MATERIALS AND METHODS

### General information

A total of 20 patients within 41-65 years old, who had osteonecrosis of the jaw, but had no cancer or osteoporosis, and never had tooth extraction, were treated in our hospital from January 2016 to March 2018, and were selected as the observation group (ONJ group). The jaw tissues and blood samples of 20 healthy donors within 42-66 years were collected and assigned as the control group (NC group). Inclusion criteria: (1) clinical diagnosis of drug-induced jaw necrosis; (2) 40-70 years old; (3) surgical treatment, and no history of radiotherapy; (4) no history of cardiovascular disease or other serious diseases, no history of psychosis. Exclusion criteria: non-ZOL drug-induced jaw necrosis, no surgical treatment, and subjects without the treatment. All patients included in the study were informed about the research content and provided a signed informed consent. The enrollment of subjects was approved by the Ethics Committee of the institution of Shanghai Jiaotong University, School of Medicine.

### Methods

### Comparison of clinical experiments

(1) The necrotic jaw tissues, oral mucus and blood samples of patients in the treatment group and necrosis of jaw were collected. The healthy jaw tissues, oral mucus and blood samples in the control group were also collected. The jaw tissues were extracted with ultrasonic bone scalpel under aseptic conditions during the operation, and the oral mucus was collected using an IDEXX Oral Mucus Collection Kit.

Two groups of fasting blood samples were collected in the morning. These were fixed in room temperature for one hour, and centrifuged for 10 minutes at 1,000 r/min. Then, the supernatant was absorbed and stored in a refrigerator at -20° C.

(2) Immunohistochemical method (IHC) was used to analyze the distribution of CD11b+Gr1hi cells and γδ-T cells in the necrotic tissue of the jaw.

The slices were dewaxed in xylene, and hydrated with gradient ethanol. Then, the endogenous peroxidase was removed at room temperature for 30 minutes. Afterwards, the PBS antigen solution that contained 10% fetal bovine serum was sealed at room temperature for one hour. After three rinses, anti-CD11b + Gr1hi cells were added with the primary anti-Ly6G (Gr1) antibodies or primary anti-CD11b antibodies, respectively. Next, γδ-T cells were incubated overnight with GL3 and DX5 antibodies (diluted at 1:200). After three rinses, the cells were added with hot pepper. The root peroxidase-labeled anti-rat/rabbit universal second antibody was incubated at 37° C for 30 minutes, washed with 1× PBS for three times, stained with the DAB reaction for 5-8 minutes, dyed with hematoxylin for 10 minutes after rinsing with single steam water, transparent with 1% alcohol hydrochloride for 1-2 seconds, and blue with hot water above 90° C for three minutes. Finally, these were dried with water and ethanol for three minutes, and sealed with neutral gum. Then, the tissue staining was observed under a microscope. Five visual fields were randomly selected from each slide to calculate the percentage of positive cells in the total number of cells.

(3) Flow cytometry was used to analyze the proportion of CD11b+Gr1hi cells and γδ-T cells in oral inflammatory fluid.

CD11b+Gr1hi cells and γδ-T cells were indirectly cultured. The percentage of CD11b+Gr1hi cells was detected by flow cytometry. The single cell suspension of the controls, CD11b+Gr1hi, and γδ-T cells was 5×10^5^ cells, respectively, and the concentration of the bovine serum protein PBS solution was 10 g/L. The CD11b + Gr1hi cells were stained with Ly6G (Gr1) and CD11b antibodies, and γδ-T cells were stained with GL3 and DX5 antibodies, while was not performed for the control group. The positive rates of CD11b+Gr1hi cells and γδ-T cells were determined by computer analysis.

(4) ELISA was used to analyze the content of inflammatory factors TNF-α, IL-1β, IFN-γ, CCL3, CCL4, IL-12 and IL-13 in blood.

The detection was carried out according to the instructions of the kit. The standard curve was made by the ratio of standard substance, and incubated at 37° C for 60 minutes at 100 μL per hole in the microporous plate. The absorbance value was determined at 450 nm using the enzyme labeling instrument after washing the plate, and the steps of biotin antibody, enzyme conjugates, chromogenic reagents and termination of the reaction.

### Experimental comparison of rats

(1) Establishment of the animal model: A total of 40 male Sprague Dawley (SD) rats (specific pathogen free [SPF] grade, 200-220 g, 10 weeks old) were selected to establish the animal model of jaw necrosis. These rats had not receive tooth extraction, and recorded as the observation group (ONJ group). The other 10 rats were assigned as the control group (NC group). The feeding environment was 12 hours of cyclic light, at a temperature of 22-25° C, with free diet. Normal saline (0.1 mL/kg) was intraperitoneally injected in the NC group. Rats in the ONJ group received intraperitoneal injection of zoledronate sodium, at 0.2 mg/kg per week, which was divided into three sessions, with each injection at 66 μL/kg, and a continuous injection for 12 weeks. At week nine, all rats were intraperitoneally injected with 3% sodium pentobarbital (0.15 mL/kg). The first molar of the left mandible was extracted under general anesthesia. The operation strictly followed the aseptic principle, in order to avoid the infection of the extraction wound. Oral cleaning was performed with 0.9% chlorhexidine before and after the operation. After the last injection of zoledronate sodium for three days at the 12^th^ week, the blood samples of the animal model were collected, and the left mandible of rats was completely removed under excessive anesthesia. Then, 4% formaldehyde solution was immobilized for 24 hours for histological examination.

(2) The distribution of CD11b+Gr1hi cells and γδ-T cells in the necrotic tissue of the jaw was analyzed by IHC.

(3) The blood samples obtained from the animal models were collected before the treatment, and at each week after treatment. The proportion of CD11b+Gr1hi cells and γδ-T cells was analyzed by flow cytometry, and the levels of inflammatory factors TNF-a, IL-1β, IFN-γ, CCL3, CCL4, IL-12 and IL-13 in the blood samples were measured by ELISA.

(4) Western blot was used to analyze the expression of CTSK, FGFR3, NF-κB and ERBB2IP.

The total protein of rat osteonecrosis was extracted by cell lysis. Then, the Lowry protein was quantified, SDS-PAGE gel electrophoresis was performed, nitrocellulose membrane transfer was conducted, antibody incubation, and enzyme staining. The Tanon-2500R full automatic digital gel imaging system was used for the imaging, and the Scion Image software was used for the analysis of the protein electrophoresis band gray value.

### Statistical method

SPSS 19.0 software was used to analyze the data. The measurement data was expressed in mean ± standard deviation (mean ± SD), and the comparison between groups was expressed in the form of unpaired *t*-test. The counting data was expressed in the form of percentage (%), and the comparison between groups was analyzed by chi-square test. The statistical analysis revealed a significant difference of *P*<0.05.
